# Clique-like Point Cloud Registration: A Flexible Sampling Registration Method Based on Clique-like for Low-Overlapping Point Cloud

**DOI:** 10.3390/s24175499

**Published:** 2024-08-24

**Authors:** Xinrui Huang, Xiaorong Gao, Jinlong Li, Lin Luo

**Affiliations:** School of Physical Science and Technology, Southwest Jiaotong University, Chengdu 610031, China; xinruih497@gmail.com (X.H.); jinlong_lee@126.com (J.L.); happyluolin@vip.163.com (L.L.)

**Keywords:** 3D sensor perception, point cloud registration, clique-like

## Abstract

Three-dimensional point cloud registration is a critical task in 3D perception for sensors that aims to determine the optimal alignment between two point clouds by finding the best transformation. Existing methods like RANSAC and its variants often face challenges, such as sensitivity to low overlap rates, high computational costs, and susceptibility to outliers, leading to inaccurate results, especially in complex or noisy environments. In this paper, we introduce a novel 3D registration method, CL-PCR, inspired by the concept of maximal cliques and built upon the SC^2^-PCR framework. Our approach allows for the flexible use of smaller sampling subsets to extract more local consensus information, thereby generating accurate pose hypotheses even in scenarios with low overlap between point clouds. This method enhances robustness against low overlap and reduces the influence of outliers, addressing the limitations of traditional techniques. First, we construct a graph matrix to represent the compatibility relationships among the initial correspondences. Next, we build clique-likes subsets of various sizes within the graph matrix, each representing a consensus set. Then, we compute the transformation hypotheses for the subsets using the SVD algorithm and select the best hypothesis for registration based on evaluation metrics. Extensive experiments demonstrate the effectiveness of CL-PCR. In comparison experiments on the 3DMatch/3DLoMatch datasets using both FPFH and FCGF descriptors, our Fast-CL-PCRv1 outperforms state-of-the-art algorithms, achieving superior registration performance. Additionally, we validate the practicality and robustness of our method with real-world data.

## 1. Introduction

With the advancement and widespread application of 3D sensing technology, various sensor types, including laser scanning [1], structured light [2], and stereo cameras [3], have reached maturity. As a result, the use of 3D point clouds in applications such as localization [4] and 3D reconstruction [5] has become increasingly important. However the point cloud data captured by different sensors often exhibit variations in orientation and position, leading to misalignments between point clouds. Consequently, 3D point cloud registration (PCR) has emerged as a fundamental problem in 3D computer vision [6], serving as a prerequisite for numerous downstream tasks. The primary objective of PCR is to determine a six-degrees-of-freedom (6-DOF) transformation that accurately aligns point clouds from neighboring stations [7]. In practical scenarios, point cloud scans, especially of natural scenes, may not always guarantee sufficient overlap. This is particularly true in cases like sparse vehicular lidar scans or when occlusions, temporal differences, and interruptions occur [8]. Low overlap in such scenarios often results in an increase in outliers, posing a significant challenge for PCR.

To address this challenge, two main approaches are generally utilized: designing more discriminative 3D feature descriptors and developing effective outlier rejection methods. In terms of 3D feature descriptors, some approaches [9,10,11,12,13,14] utilize deep learning techniques to extract richer features from point clouds or to develop more discriminative feature description. On the other hand, traditional methods [15,16,17,18,19] focus on characterizing features based on the intrinsic information of the point cloud itself. Among outlier rejection methods, RANSAC [20] guides the registration process through a straightforward iterative sampling strategy. This approach was pioneering in guiding registration by sampling expected inliers. However, despite its simplicity and efficiency, the performance of RANSAC-based methods significantly deteriorates as the outlier ratio increases, leading to higher computational demands [21]. Additionally, deep learning-based methods often require large datasets for training and may lack generalization capabilities across different datasets [22]. As a result, achieving accurate registration in the presence of a high number of outliers and across varying datasets remains a considerable challenge.

In this paper, we propose CL-PCR, a novel registration method inspired by the concept of maximal cliques [22] and built upon the SC^2^-PCR [23] framework. Our method follows these key steps: first, we construct a spatial compatibility matrix using the SC^2^-PCR method; second, we generate clique-like subsets through our proposed approach; third, these clique-like subsets are processed using the SVD algorithm to generate transformation hypotheses; and finally, we apply evaluation metrics to select the optimal model. Overall, our main contributions are as follows:

(1) Proposed CL-PCR Method: We introduce a new sampling hypothesis generation method called CL-PCR. Our clique-like approach, inspired by the concept of maximal cliques, relaxes the constraint of a fixed number of samples compared to SC^2^-PCR. This flexibility allows us to extract more reliable hypotheses from smaller clique-like subsets. The effectiveness and practicality of our approach are demonstrated in subsequent experiments.
(2) Clique-Like Sampling Enhancement Methods: Building on our clique-like model, we propose two sampling enhancement methods. These methods address the problem of outlier penetration and leverage the consensus information from deep-sampling clique-like subsets to generate more reliable hypotheses.
(3) Extensive Experimental Validation: We conduct comprehensive experiments to demonstrate the effectiveness of our sampling hypothesis generation method. We adopt the FS-TCD metric [24] as a secondary evaluation metric, in combination with three other metrics: IC, MAE, and MSE. This robust evaluation framework allows for detailed analysis and model selection for hypothesis generation.
(4) Superior Performance and Practicality: Our method outperforms state-of-the-art algorithms, achieving the highest registration recall on the 3DMatch and 3DLoMatch datasets using both FPFH and FCGF descriptors. Additionally, we demonstrate the practicality and robustness of our approach on real-world train datasets.

## 2. Related Works

### 2.1. Three-Dimensional Feature Descriptors

In the task of 3D point cloud registration (PCR), correspondence-based methods are well-established and reliable approaches, primarily relying on feature descriptors with high discriminative ability.

Deep learning-based feature descriptors. These descriptors are typically trained using convolutional networks and large datasets to generate representative and distinctive features. PointNet [9], for instance, extracts features using a multi-layer perceptron (MLP) in a simple yet effective structure. More advanced methods include 3DMatch [10] and 3DSmoothNet [11], which utilize deep convolutional neural networks (CNNs) as feature extractors. SpinNet [12] introduces a 3D cylindrical convolutional network (3DCCN) for feature mapping, while DGCNN [13] incorporates the EdgeConv aggregation module to capture local geometric features. FCGF [14] connects points as sparse tensors and constructs full convolutional networks for feature extraction using the Minkowski Engine [25]. Despite the strong performance of these deep learning-based methods, they typically require large amounts of training data and are susceptible to overfitting and poor generalization across different datasets.

Traditional feature descriptors. These descriptors generally encode the domain coordinates, normals, and other information of the query points in the point cloud. Spin Image [15] encodes the local surface shape as a descriptor and forms rotationally invariant features by projecting the local neighborhoods of each point onto a two-dimensional image. The 3DSC [16] extends the shape context from 2D to 3D, describing point cloud features by computing the distribution of local neighborhoods of points in a spherical coordinate system. SHOT [17] describes the point cloud by computing the normal vectors information in the subspace of points to statistically encode histograms, providing better robustness and descriptive capability. PFH [18] encodes information such as normal angles and distances between pairs of points in the form of statistical histograms, providing a rich geometric description, despite its high computational complexity. FPFH [19] reduces computational complexity by simplifying the computation of point-pair features and replacing PFH with the weighted result of SPFH, making it more efficient in practical applications.

### 2.2. Outlier Rejection

After feature matching, typical correspondence-based registration methods can directly obtain the six-degrees-of-freedom (6-DOF) transformation using Singular Value Decomposition (SVD). However, in scenarios with low overlap ratios, the feature matching process often contains a large number of incorrect correspondences, making outlier rejection critical.

Learning-based outlier rejection. Some approaches integrate outlier rejection as a module within their overall network architecture. For instance, Predator [26] and GeoTransformer [27] propose attention modules to better differentiate between inliers and outliers, while BUFFER [28] addresses the problem of low overlap ratio by designing an inliers generator. Other methods treat outlier rejection as an independent classification problem. DGR [29], 3DRegNet [30], and PointDSC [31] achieve outlier rejection by designing independent classification networks to predict the confidence level of inliers.

Traditional outlier rejection. The sampling hypothesis generation model of RANSAC [20] and its variants [32,33] remains the dominant approach for outlier rejection. These methods select expected inliers for registration by randomly sampling from the initial correspondence set, iteratively generating hypotheses, and evaluating them to select the best one for registration. However, RANSAC and its variants often struggle to maintain high accuracy in scenarios with extremely high outlier ratios. Rather than optimizing the registration function directly, some alternative approaches focus on finding the subset of correspondences that are pairwise consistent [34]. For example, PMC [34] describes the correspondence set as an undirected graph and searches for its maximum clique to find the expected inliers directly, though its branch-and-bound search is impractical [35]. TEASER [36] also employs maximum clique and geometric constraints for outlier pruning. SC^2^-PCR [23] distinguishes between inliers and outliers using a second-order metric, searching for a consistent set of points from seed points for pairwise registration. MAC [22] combines a relaxed maximal clique strategy with a second-order metric based on [23] to fully consider the possibilities of each hypothesis, demonstrating that even just three points can potentially become the best registration inliers.

## 3. Methods

### 3.1. Problem Formulation

**Figure 1:**
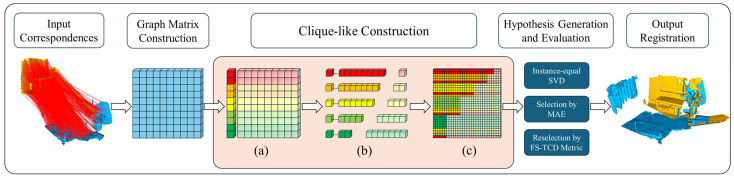
Framework of CL-PCR. First, construct a graph matrix from the initial correspondence set. Second, select consistent sets from the graph matrix to form clique-like subsets of different sizes. Third, generate hypotheses from the clique-like subsets and select them by the evaluation metrics. Fourth, select the best hypothesis for the registration. (**a**): Identifying the initial node. (**b**): Finding the filling nodes. (**c**): Sampling enhancement.

For two point clouds that need to be aligned, we denote the source point cloud as $P=\left\{p_{i}\in R^{3}\;\right|\;i=1,\ldots ,N_{p}\}$ and the target point cloud as $Q=\left\{q_{i}\in R^{3}\;\right|\;i=1,\ldots ,N_{q}\}$. We firstly extract their local features by using either traditional or learning-based feature descriptors. Feature matching of the extracted features yields an initial correspondence set $C=\left\{c\;|\;c=(p,q)\right\}$. Next, we characterize the correspondences c with different subsets of samples as clique-likes and use the clique-likes to search for potential inliers to guide the registration. The framework of our method is shown in Figure 1.

### 3.2. Graph Matrix Construction

Graph spaces can describe the relationships between correspondences more accurately than Euclidean spaces [22]. However, constructing an actual graph can be extremely time-consuming, especially as the number of correspondences increases. To address this, our method does not construct a real graph but instead follows [23] and utilizes two approaches to construct the compatibility graph matrix.

First-order matrix. The first-order matrix $\left(FM\in R^{N\times N}\right)$ is represented by a rigid Euclidean distance constraint. The difference in spatial distances between the correspondence pair $\left(c_{i},c_{j}\right)$ can be expressed as follows:

(1) $$Dist\left(c_{i},c_{j}\right)=\left|∥p_{i}-p_{j}∥-∥q_{i}-q_{j}∥\right|$$

The compatibility between them can be expressed as a truncated distance measure for nonlinear mappings:

(2) $$cmp\left(c_{i},c_{j}\right)=\max(1-\frac{{Dist\left(c_{i},c_{j}\right)}^{2}}{\tau^{2}},0)$$

where τ is the inlier threshold parameter. This score metric helps to better filter the inliers from the outliers. We use this score to represent the compatibility relationship between the correspondence pair $\left(c_{i},c_{j}\right)$ and store it in the $FM\left(i,j\right)$ element of the FM, which is symmetric. Hence, $FM\left(j,i\right)=FM\left(i,j\right)$.

Second-order matrix. The metric of the second-order matrix $(SM\in R^{N\times N}$), proposed by [23], aims to further distinguish between inliers and outliers from globally compatible relations. The FM is first re-corrected to measure the spatial distance difference directly using the inlier threshold parameter τ and reduced to a boolean value to re-derive the hard first-order matrix $\left(\bar{FM}\in R^{N\times N}\right)$:

(3) $$\bar{FM}(i,j)=\left\{\begin{array}{c} 1;Dist\left(c_{i},c_{j}\right)\leq \tau \\ 0;Dist\left(c_{i},c_{j}\right)>\tau \end{array}\right.$$

The SM can then be computed using the following equation:

(4) $$SM=\bar{FM}⊙(\bar{FM}\times \bar{FM})$$

where ⊙ denotes element-wise multiplication.

In comparison, SM is a more efficient metric than FM and can significantly differentiate between inliers and outliers. In Section 4.5, we compare these two methods of graph matrix construction.

### 3.3. Clique-like Construction

Given an undirected Graph=(Vertex,Edge) and a $Clique=(Vertex^{\prime }\in Vertex,Edge^{\prime }\in Edge)$ within the graph, the clique is a subset where every pair of nodes $Vertex^{\prime }$ is connected by an $Edge^{\prime }$. This indicates that all nodes in the clique are in mutual consensus. The key idea behind clique-based registration is to identify a clique where this consensus suggests potential inliers. In our work, we construct clique-like using a fast approximation method based on the inherent properties of cliques. The clique-like we construct can simulate the function of mutually constrained subsets of maximal cliques. We define a ${Clique\_like}_{n\;}=\{c_{i}\in C|i=1,\ldots ,N_{n}\}$ with n nodes, where C is the initial correspondence set, $c_{i}$ represents the clique-like nodes, and $N_{n}$ denotes the number of nodes. Our goal is to construct clique-likes that are expected to satisfy the consistency for all nodes.

The construction process of our clique-like structures is straightforward, involving two main steps: identifying the initial node and finding the filling nodes. As illustrated in the orange section of Figure 1, we begin by constructing a more relaxed FM and applying the leading eigenvector algorithm to calculate evaluation scores for each correspondence in relation to the others based on global information. This global information is embedded in an eigenvector ($V\in R^{N\times 1}$), which represents the potential global compatibility score for each correspondence. Each row’s correspondence is then used as the initial node in the clique-like. We classify all the initial nodes into five categories based on their compatibility scores. As shown in Figure 1a, the rows are color-coded by score, where a higher score indicates a greater likelihood that the node is connected to other nodes. Next, we populate the nodes with information from the SM to better distinguish between inliers and outliers. The leading eigenvector algorithm is once again employed to calculate the evaluation score of each initial node relative to other correspondences in the SM. This enables us to rank and select the remaining $N_{n}-1$ correspondences with the highest consistency as neighbor padding. In Figure 1b, the initial nodes are shown to connect with varying numbers of nodes to complete the filling of the clique-like subsets.

**Figure 2:**
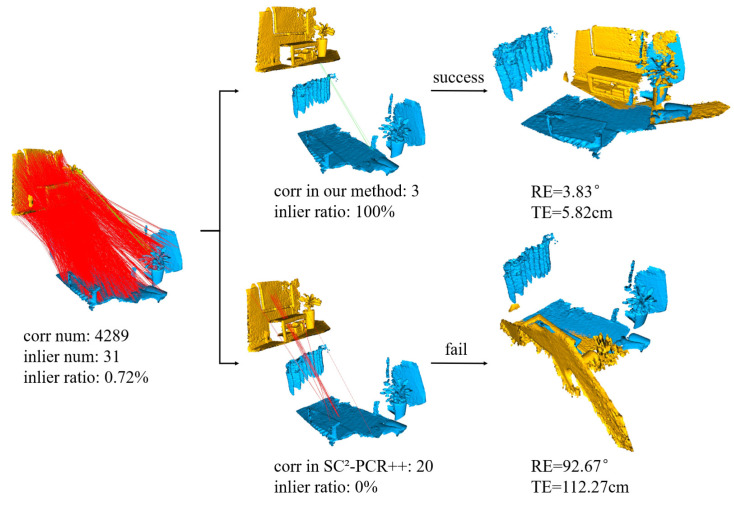
Comparison of our method with the SC^2^-PCR method in the case of extremely low inlier ratio within point cloud pairs.

Maximal cliques registration fully explores the potential combinations of individual correspondence subsets. Drawing inspiration from the maximal cliques, we relax certain constraints and uncover more potential information by designing clique-like structures with varying numbers of nodes. As shown in Figure 2, compared to SC^2^-PCR++, our method allows fewer consensus sets to participate in hypothesis generation. This capability enables us to successfully find the correct consensus subset, even in extreme cases where the inlier ratio is less than 1%. In this example, our method performs well in challenging situations with as few as three sets of correspondences. This approach is particularly effective in uncovering subtle and difficult-to-detect information.

However, Ref. [37] revealed the problem of outlier correspondence penetration in maximum cliques. This means that the subsets generated by the maximum cliques may still include some outliers, which could negatively impact the registration process. Similarly, the sampling subset method used in SC^2^-PCR, which samples a fixed number of correspondences, is also susceptible to this problem. Although our clique-like method offers greater flexibility in selecting subsets compared to SC^2^-PCR, it is not immune to this issue. To mitigate this, we propose the following clique-like sampling augmentation strategies:

Default demotion strategy. We establish five types of clique-like structures, each with a different number of nodes. Unlike the typical construction process discussed earlier, we acknowledge that in actual maximal cliques, larger cliques are fewer in number, while smaller cliques are more common, resembling a pyramid structure, as shown in Figure 1c. To augment our approach, we employ a default demotion strategy, where we randomly demote some of the initial nodes of the larger clique-like subsets into smaller ones by reducing their neighborhood node padding. This strategy aims to ensure that each clique-like structure contains the most probable inliers, even if it means sacrificing some inliers to address the issue of outlier correspondence penetration. However, this strategy has a drawback: it may truncate large subsets filled with inliers, reducing them to smaller subsets that contain only a few inliers. To counteract this limitation, we propose a second enhancement strategy.

Safe demotion strategy. In this strategy, we continue to demote large-sized clique-like structures into smaller ones. However, to avoid the risk of mistakenly truncating subsets that consist entirely of inliers, we implement a safer demotion approach. This involves randomly demoting the initial nodes while also retaining them in their original classification. This method allows for further truncated sampling to remove outliers from clique-like structures that suffer from outlier penetration, while ensuring that clique-like structures already filled with valid inliers are not mistakenly removed.

A comparison of the default clique-like construction method and the two enhanced construction methods is provided in Section 4.5.

### 3.4. Hypothesis Generation and Evaluation

Each set of clique-like represents a group of expected consistent inliers. By applying the SVD algorithm, we can obtain a set of 6-degrees-of-freedom positional hypotheses.

Instance-equal SVD. SVD is the usual method for transformation, where “instance-equal” implies that every correspondence involved in the computation is equally weighted.

Weighted SVD. Recent methods, including those proposed in [27,29] and SC^2^-PCR [23], have explored the application of weights to the correspondence set to better guide the registration process. Typically, these weights are derived from the eigenvector of the compatibility matrix. Following the SC^2^-PCR approach, we compute the eigenvector of the local SM for each set of clique-like structures and use it as the weights for the weighted SVD.

After generating the hypotheses using SVD, the optimal hypothesis is selected based on evaluation metrics. We consider several popular hypothesis evaluation metrics from RANSAC [38], including inlier count (*IC*), mean average error (*MAE*), and mean square error (*MSE*). The evaluation formula for the *k*-th hypothesis is as follows:

(5) $${IC}_{k}=\sum_{i=1}^{N}\left[∥R_{k}p_{i}+t_{k}-q_{j}∥<\tau \right]$$

(6) $${MAE}_{k}=\sum_{i=1}^{N}\left[\frac{\left|\left\|R_{k}p_{i}+t_{k}-q_{j}\right\|-\tau \right|}{\tau }<\tau \right]$$

(7) $${MSE}_{k}=\sum_{i=1}^{N}\left[\frac{{\left(∥R_{k}p_{i}+t_{k}-q_{j}∥-\tau \right)}^{2}}{\tau^{2}}<\tau \right]$$

where N is the number of putative correspondences, ${(p}_{i},q_{j})$ represents a pair of correspondences, $\left[\cdot \right]$ denotes the Iverson bracket (which returns 1 if the condition inside the parentheses is true, and 0 otherwise), and τ is the conditional threshold for judging the inliers. $R_{k}$ and $t_{k}$ are the rotation and translation matrices of the *k*-th hypothesis. When both are correct estimates, the ${IC}_{k}$ should be close to the number of inliers. The metrics ${MAE}_{k}$ and ${MSE}_{k}$, which are weighted versions of ${IC}_{k}$, are expected to reflect the registration differences more effectively and accurately. However, a recent study [24] noted that in some scenarios with a low overlap ratio, the inlier counts might be small when correctly registered. To address this, we also incorporate the feature and spatial consistency constrained truncated chamfer distance (*FS* − *TCD*) as an additional evaluation metric. This metric is formulated as follows:

(8) $${FS-TCD}_{k}=\sum_{i=1}^{N^{\prime }}\left[(\underset{H_{\mathrm{ij}}=1}{\min}\left\|R_{k}p_{i}+t_{k}-q_{j}\right\|)<\eta \right]$$

where $N^{\prime }$ is the number of putative correspondences further filtered after spatial consistency assessment. H is the relaxed feature matching relationship matrix, where $H_{ij}=1$ indicates that ${(p}_{i},q_{j})$ must be consistent in feature metric. The meaning of this formula is that for each $p_{i}$, we find the closest $q_{j}$ in the target point cloud Q after applying the transformations $R_{k}$ and $t_{k}$. We then count the number of inlier pairs whose error is less than the threshold η. The count is valid if and only if the pair ${(p}_{i},q_{j})$ is consistent in both feature and space. A more detailed derivation can be found in [24].

The more advanced and effective the evaluation metrics are, the more useful the multiple hypotheses of our clique-like can be. The performance of each evaluation metric is discussed in Section 4.5.

## 4. Experimental Section

### 4.1. Experimental Setup

Datasets. We validate our method using three datasets: the indoor datasets 3DMatch and 3DLoMatch, and the outdoor dataset KITTI. The 3DMatch dataset contains 1672 pairs of point clouds, while the more challenging 3DLoMatch dataset contains 1781 pairs with an overlap ratio of only 10% to 30%. For the KITTI dataset, we follow [22,23] and use 555 pairs of point clouds for testing.

Evaluation Criteria. We primarily report the registration recall (RR) within a certain error range as the main evaluation criterion. Additionally, following [29], we measure the registration effectiveness using the rotation error (RE) and translation error (TE). For the 3DMatch and 3DLoMatch datasets, registration is considered successful when RE ≤ 15° and TE ≤ 30 cm. For the KITTI dataset, registration is considered successful when RE ≤ 5° and TE ≤ 60 cm. Furthermore, following [29], since failed registrations can cause significant errors, we present both RE and TE in the experimental tables as errors from successfully registered pairs only.

Implementation Details. All input correspondence sets are generated by traditional FPFH or learning-based FCGF descriptors. Following our clique-like construction methods described in Section 3.3, we set up five types of clique-like and find initial nodes. We refer to [22] to set the node of our smallest clique-like to 3, and refer to [23] to set the max node to 20. The overall five types of clique-like sizes are sequentially decreased as 20, 15, 10, 5, and 3. Subsequently, we implement the clique-like sampling enhancement method proposed in Section 3.3, where the node demotion ratio is set to 0.5. Finally, hypotheses are generated from the correspondences in each clique-like using instance-equal SVD, and they are evaluated by MAE and FS-TCD. All the experiments are conducted on a machine with an Intel i9 12900k CPU (Intel Corporation, Santa Clara, CA, USA) and a single NVIDIA RTX3090 (NVIDIA, Santa Clara, CA, USA).

### 4.2. Results on 3DMatch Dataset

In this experiment, we first test the 3DMatch dataset. As shown in Table 1, our CL-PCR outperforms all other compared methods in the correspondences generated using the traditional descriptor FPFH. For the RR, which is the most important evaluation metric in registration, our method shows a 4.38% improvement over the state-of-the-art MAC algorithm, a 1.30% improvement over SC^2^-PCR++, and a 10.91% improvement over the deep learning method PointDSC. It is important to note that the RE and TE calculations are derived from the results of successful registrations. This strategy can lead to methods with high RR tending to introduce larger errors, as they include more difficult-to-align data in their error calculations than methods with low RR [23]. Nevertheless, our RE and TE remain relatively substantial. When using the FCGF descriptor, the correspondence inlier ratio improves compared to FPFH, as expected, leading to better performance across all correspondence-based comparison methods. As shown in Table 1, our method still achieves strong performance, with an RR slightly lower than SC^2^-PCR++ but 0.18% higher than MAC. Notably, in subsequent experiments with the proposed optimization models, our Fast-CL-PCRv1 achieves the highest registration recall.

### 4.3. Results on 3DLoMatch Dataset

To further demonstrate the robustness of our method, we test it on the low-overlap scene dataset 3DLoMatch. Following [22], we use FPFH and FCGF as feature descriptors to generate correspondences. The results are shown in Table 2. From the data in the table, our method also performs well in the benchmark. Notably, our Fast-CL-PCRv1 performs second only to our original CL-PCR with FPFH, while outperforming all other algorithms with FCGF.

**Figure 3:**
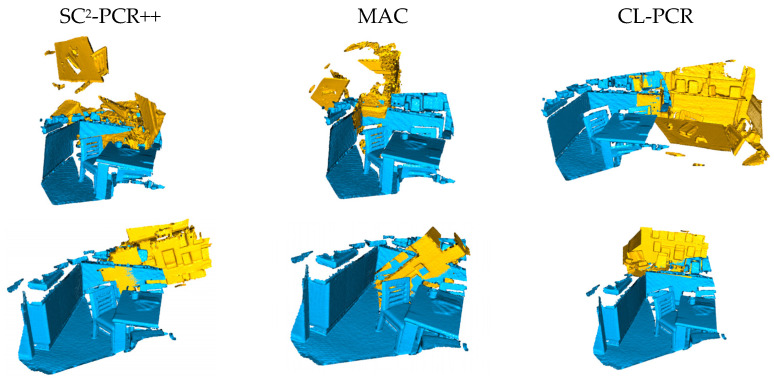

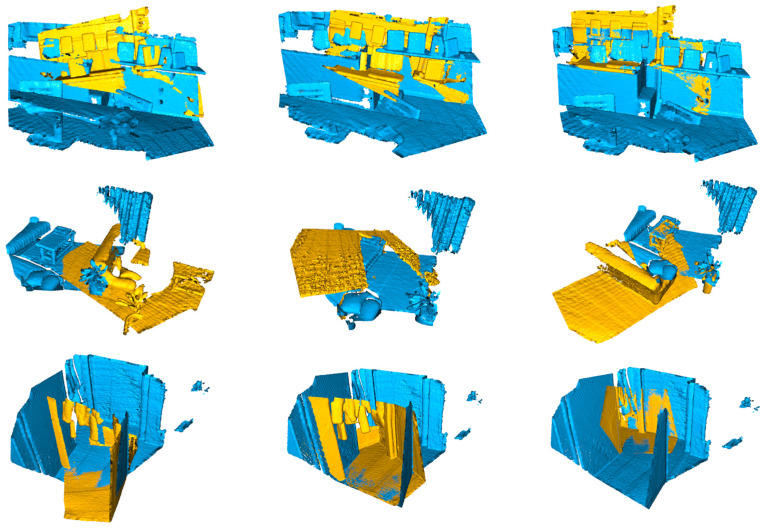
Qualitative comparison on low-overlapped 3DLoMatch dataset. From left to right are SC^2^-PCR++ [24], MAC [22], and ours.

In addition, we present some qualitative results from the 3DLoMatch dataset, which features low overlap ratios. As shown in Figure 3, we compare our method with SC^2^-PCR++ and MAC, showcasing visual registration outcomes for challenging data. The results clearly demonstrate that our method is capable of accurately registering point cloud pairs even in scenarios with very low overlap.

### 4.4. Results on KITTI Dataset

In this experiment, we evaluate our method using the outdoor dataset KITTI, and the results are shown in Table 3. When FPFH is used for generating correspondences, our method achieves the second-highest RR, just behind SC^2^-PCR++. Compared to MAC, our method matches the RR but results in lower rotation and translation errors. When using FCGF for generating correspondences, our method achieves the highest RR, equaling SC^2^-PCR++. This consistency across different feature descriptors highlights the robustness of our approach.

The results from both indoor and outdoor datasets (3DMatch, 3DLoMatch, and KITTI) highlight the robustness and versatility of our method across various application scenarios. These findings underscore the excellent capability of our method in generating accurate registrations across different environments.

### 4.5. Analysis Experiments

In this section, we conduct and analyze the ablation experiments of our method on the 3DMatch and 3DLoMatch datasets using the FPFH descriptor. We compare several algorithms introduced in Section 3. To avoid random errors caused by the clique-like sampling enhancement method, we ensure consistency in the inputs for each set of ablation experiments by sequentially selecting the initial nodes. This approach allows us to focus on the impact of different strategy choices rather than the variability introduced by random node selection. The results of the ablation experiments for different combinations of methods are presented in Table 4 and Table 5. Additionally, we analyze the selection rate and recall rate of the five types of clique-like in our method, with the findings shown in Table 6. To further improve efficiency, we propose three optimization models, with their results displayed in Table 7 and Table 8. Lastly, we evaluate the upper performance limits of our method compared to the SC^2^-PCR++ method, as illustrated in Table 9.

Clique-like construction methods. We test the three clique-like construction methods mentioned in Section 3. As shown in Table 4 and Table 5 (rows 1 to 3), the RR is highest with the clique-like sampling enhancement method using the safe demotion strategy. This method shows an improvement of 0.56% on the 3DMatch dataset and 0.17% on the 3DLoMatch dataset compared to the normal construction method. These results demonstrate that demoting some nodes to smaller clique-like subsets, while retaining the original larger subsets, can effectively address the issue of outlier penetration and enhance overall registration performance. In contrast, the data enhancement method using the default demotion strategy is less effective than the non-enhancement method, indicating that the forced pruning of certain subsets can be counterproductive.

Choices of graph matrix construction. In this paper, we use the graph matrix twice. The first usage is for initial node sorting, where we default to the more relaxed first-order matrix (FM). The second usage is during the construction of clique-like, where we compare the effectiveness of different graph matrices. As shown in Table 4 and Table 5 (rows 3 and 4), employing the second-order matrix (SM) to construct the graph matrix results in a 2.47% improvement in RR on the 3DMatch dataset and a 6.85% improvement on the 3DLoMatch dataset compared to using FM. This enhancement suggests that SM is more effective at distinguishing between inliers and outliers when identifying neighboring nodes for the initial node, thereby facilitating the construction of more robust clique-likes and leading to better registration outcomes.

Weighted SVD vs. instance-equal SVD. We compare the performance of instance-equal SVD and weighted SVD, as shown in Table 4 and Table 5 (rows 3 and 8). Our method using instance-equal SVD shows a 0.3% higher RR on the 3DMatch dataset and a 0.4% higher RR on the 3DLoMatch dataset compared to using weighted SVD. Although weighted SVD is widely employed in SC^2^-PCR and other state-of-the-art methods, the unweighted SVD approach proves more effective for hypotheses generated by small clique-like subsets with fewer nodes. This approach helps to minimize the interference of global information, thereby facilitating the generation of correct hypotheses.

Evaluation metrics selection. We compare three model selection methods: IC, MAE, and MSE, along with the recently proposed reselection metric FS-TCD for hypothesis evaluation of our method. As shown in Table 4 and Table 5 (rows 3 and 7), our method achieves the highest performance with MAE + FS-TCD. This combination of reselection by FS-TCD improves RR by 4.87% on the 3DMatch dataset and 4.27% on the 3DLoMatch dataset, demonstrating that advanced metrics are more effective at evaluating hypotheses generated by fewer but more reliable nodes in our clique-like method. When using reselection as depicted in Table 4 and Table 5 (rows 3, 5, and 6), MAE results in a 0.99% higher RR than MSE on the 3DMatch dataset and 0.23% higher on the 3DLoMatch dataset. Additionally, MAE yields the same RR as IC on the 3DMatch dataset while reducing the rotation error (RE) by 0.02° and the translation error (TE) by 0.02 cm. On the 3DLoMatch dataset, MAE outperforms IC in RR by 0.96%.

Clique-like model selection analysis. To further assess the effectiveness of our method across different clique-like sizes, we evaluate the selection and recall of five differently sized clique-like structures on the 3DMatch and 3DLoMatch datasets using the FPFH and FCGF descriptors. The results are summarized in Table 6. The AS statistic shows that the five clique-like models with varying node counts contribute evenly to the final model selection. This indicates that our clique-like models are both effective and meaningful, with no undue reliance on any single size. In terms of the AP statistic, we analyze the recall performance for each clique-like model. Notably, the model consisting of only three nodes achieves the highest recall. This finding highlights the effectiveness of our subset mining process in identifying valuable hypotheses.

Model optimization analysis. In this experiment, we optimize several components of CL-PCR to enhance model efficiency. FS-TCD is particularly suitable for cases where the inlier count metric becomes unreliable, especially with low point cloud pair overlap ratios. We define that an inlier count of fewer than 100 is no longer entirely reliable. Additionally, smaller clique-likes are more effective for extreme cases, as shown in Figure 2, while using all hypotheses can be time-consuming in simpler scenarios. Thus, we propose that when the inlier count is less than 30, smaller clique-likes are preferable for deeper mining. Based on this observation, we optimize the model by reducing resource allocation for simpler pairs to improve efficiency. We measure the inlier count using the highest MAE score of the hypotheses. The impacts of these optimizations are presented in Table 7 and Table 8. Furthermore, we introduce three optimization models through different algorithms, significantly reducing computation time and enhancing registration efficiency without compromising accuracy.

Performance upper bound analysis. Previous experiments indicate that while FS-TCD is a highly advanced metric, the registration recall (RR) may increase after pruning, suggesting it is not always the best metric for model selection. In this section, we assess the performance upper bound of our method under ideal conditions. We define ideal evaluation metrics as those allowing a pair of point clouds to be correctly aligned. This helps evaluate the performance of our method against SC^2^-PCR++ under upper bound constraints.

We compare different thresholds for the number of correct hypotheses to gauge the reliability of our method relative to SC^2^-PCR++. The results, shown in Table 9, demonstrate that our method outperforms SC^2^-PCR++ at all tested thresholds. Specifically, in the success-1 experiment, our method achieves a registration recall of 96.00% and 98.34% on the 3DMatch dataset using the FPFH and FCGF descriptors, respectively, surpassing SC^2^-PCR++ by 4.5% and 1.54%. On the 3DLoMatch dataset, our method achieves 70.19% and 87.37% recall with FPFH and FCGF, respectively, exceeding SC^2^-PCR++ by 20.84% and 10.33%. These results indicate that our method is highly effective in generating correct hypotheses for most point clouds in the 3DMatch dataset. For the challenging 3DLoMatch dataset with low overlap, our method achieves up to 70.19% recall with FPFH and an even higher 87.37% with FCGF. Although our model has not yet achieved the ultimate upper limit as defined by the current evaluation metrics, the success-1 results demonstrate that our method has a higher performance ceiling. As evaluation metrics continue to improve, our model is poised to achieve even better performance.

### 4.6. Real Data Experiments

**Figure 4:**
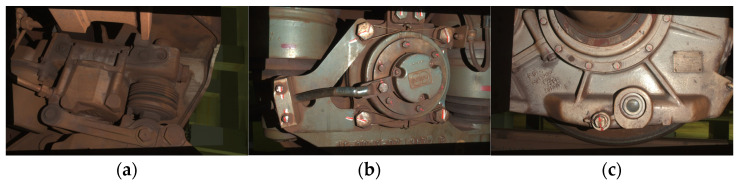
Three different parts from the train: (**a**–**c**).

**Figure 5:**
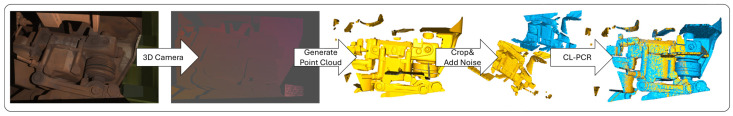
The workflow process.

In this experiment, we use three real point cloud datasets collected from a 3D laser scanner equipped with a three-million-pixel industrial camera. These datasets capture various object parts, as shown in Figure 4. The workflow for processing these datasets is illustrated in Figure 5. The process begins with the 3D camera capturing the object’s 3D information, which is then converted into point cloud format. To increase complexity, we introduce cropping and noise into the point cloud data. Following this, we downsample the point clouds using a 1 cm voxel size and extract features with the FPFH descriptor to generate correspondences. Finally, we perform the registration using our Faster-CL-PCR method.

**Figure 6:**
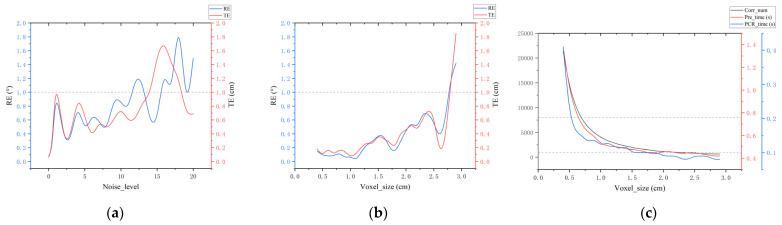
(**a**) The influence of noise level on RE and TE; (**b**) the influence of voxel size on RE and TE; and (**c**) the influence of voxel size on Corr_num, Pre_time, and PCR_time.

In this section, we compare the registration performance of our method with the state-of-the-art method SC^2^-PCR++ on real datasets with varying overlap rates. The results of this comparison are presented in Table 10. Additionally, we investigate the impact of different sampling degrees and noise levels on registration performance. The results of these investigations are illustrated in Figure 6.

Effect of different overlap rates on results. To further validate the effectiveness and robustness of our method in scenarios with low overlap rates, we test overlap rates ranging from 0.1 to 1 on real datasets. As shown in Table 10, our method performs consistently well across various overlap rates, with most errors remaining below 1. This indicates that our method is robust even in low-overlap scenarios with real data. While SC^2^-PCR++ performs admirably on public datasets, our method shows smaller errors across different overlap rates in real scenarios. Additionally, our method registers point clouds faster than SC^2^-PCR++, further demonstrating its practical advantages.

PCR processing capability of our algorithm. To further analyze the processing capability of our algorithm on real data point clouds, we focus on parts (a) from Figure 4 with an overlap rate of 0.4. We first investigate the impact of different noise levels on the algorithm. The original point cloud of the train component parts (a) contains 1,501,616 points, with a minimum bounding box size of 86 cm × 53 cm × 86 cm and an average nearest distance of 0.036 cm. Gaussian noise, with a standard deviation ranging from 0 to 20 times this level unit, was added to the point cloud. The error curve for the registration results is shown in Figure 6a. Despite substantial noise, our algorithm remains highly robust. When the noise level is below 10, the rotation error stays under 1° and the translation error remains below 1 cm (as indicated by the reference line). Notably, with no additional noise, our method achieves an error close to 0, highlighting the effectiveness of our approach even in the presence of inherent noise in the raw data.

We also investigate how the sampling rate affects the number of correspondences, time consumption, and errors. Figure 6b,c present the results of our experiments with voxel sizes ranging from 0.4 cm to 3.0 cm. As shown in Figure 6b, the error curves exhibit significant fluctuations at a 1.5 cm voxel size, but the errors remain below the reference line. Figure 6c indicates that when the number of correspondences exceeds the first reference line, both preprocessing time (Pre_time) and registration time (PCR_time) increase substantially, with total time exceeding 1 s. Combining the insights from Figure 6b,c, a 1 cm voxel size is optimal for the real datasets in this study. For correspondence number requirements, or algorithm achieves an effective balance between speed and accuracy with approximately 1000 to 8000 correspondences, which falls between the two reference lines in Figure 6c.

## 5. Conclusions

Based on the concept of maximal cliques, we propose the CL-PCR method for point cloud registration. This approach effectively mines consensus information from correspondence subsets to generate reliable hypotheses, offering a novel outlier rejection technique. It excels in handling low-overlap point cloud scenarios and can flexibly utilize the smallest clique-like subsets to enhance registration performance when the inlier ratio is very low. Compared to existing state-of-the-art algorithms, our method achieves higher registration recall with both FPFH and FCGF descriptors on the public datasets 3DMatch and 3DLoMatch. To improve practicality, we developed faster versions of the method. The evaluation on real datasets demonstrates that CL-PCR is robust across varying overlap rates and noise levels. Moreover, our method excels in both speed and precision, making it suitable for practical applications in real-world scenarios.

## Figures and Tables

**Table 1:** Registration results on 3DMatch dataset.

	FPFH			FCGF		
	RR (%)	RE (°)	TE (cm)	RR (%)	RE (°)	TE (cm)
Deep learned						
3DRegNet [30]	26.31	3.75	9.60	77.76	2.74	8.13
DGR [29]	32.84	2.45	7.53	88.85	2.28	7.02
DHVR [39]	67.10	2.78	7.84	91.93	2.25	7.08
PointDSC [31]	72.95	2.18	6.45	91.87	2.10	6.54
Traditional						
RANSAC-1M [20]	64.20	4.05	11.35	88.42	3.05	9.42
RANSAC-4M [20]	66.10	3.95	11.03	91.44	2.69	8.38
GC-RANSAC [32]	67.65	2.33	6.87	92.05	2.33	7.11
TEASER [36]	75.48	2.48	7.31	85.77	2.73	8.66
CG-SAC [33]	78.00	2.40	6.89	87.52	2.42	7.66
SC^2^-PCR [23]	83.98	2.18	6.56	93.28	2.08	6.55
SC^2^-PCR++ [24]	87.18	2.10	6.64	94.15	2.04	6.50
MAC [22]	84.10	1.96	6.18	93.72	1.89	6.03
CL-PCR	88.48	2.16	6.77	93.90	2.04	6.54
Fast-CL-PCRv1	88.48	2.18	6.84	94.21	2.04	6.53

**Table 2:** Registration results on 3DLoMatch dataset.

	FPFH			FCGF		
	RR (%)	RE (°)	TE (cm)	RR (%)	RE (°)	TE (cm)
Deep learned						
DGR [29]	19.88	5.07	13.53	43.80	4.17	10.82
PointDSC [31]	20.38	4.04	10.25	56.20	3.87	10.48
Traditional						
RANSAC-1M [20]	0.67	10.27	15.06	9.77	7.01	14.87
RANSAC-4M [20]	0.45	10.39	20.03	10.44	6.91	15.14
TEASER [36]	35.15	4.38	10.96	46.76	4.12	12.89
SC^2^-PCR [23]	38.57	4.03	10.31	58.73	3.80	10.44
SC^2^-PCR++ [24]	41.49	3.90	10.19	61.15	3.72	10.56
MAC [22]	40.88	3.66	9.45	59.85	3.50	9.75
CL-PCR	44.64	3.91	10.39	59.91	3.67	10.43
Fast-CL-PCRv1	44.13	3.92	10.27	61.93	3.75	10.48

**Table 3:** Registration results on KITTI dataset.

	FPFH			FCGF		
	RR (%)	RE (°)	TE (cm)	RR (%)	RE (°)	TE (cm)
Deep learned						
DGR [29]	77.12	1.64	33.10	96.90	0.34	21.70
PointDSC [31]	98.92	0.38	8.35	97.84	0.33	20.32
Traditional						
FGR [40]	5.23	0.86	43.84	89.54	0.46	25.72
TEASER [36]	91.17	1.03	17.98	94.96	0.38	13.69
RANSAC [20]	74.41	1.55	30.20	80.36	0.73	26.79
CG-SAC [33]	74.23	0.73	14.02	83.24	0.56	22.96
SC^2^-PCR [23]	99.64	0.32	7.23	98.20	0.33	20.95
SC^2^-PCR++ [24]	99.64	0.32	7.19	98.56	0.32	20.95
MAC [22]	99.46	0.40	8.46	97.84	0.34	19.34
CL-PCR	99.46	0.33	7.54	98.56	0.33	20.60
Fast-CL-PCRv1	99.64	0.34	7.52	98.20	0.33	20.62

**Table 4:** Ablation study on 3DMatch with FPFH descriptor. NC: Normal construction of clique-like. DD: Default demotion strategy. SD: Safe demotion strategy. RS: Reselection by FS-TCD. W-SVD: Weighted SVD.

	NC	DD	SD	FM	SM	IC	MAE	MSE	RS	SVD	W-SVD	RR (%)	RE (°)	TE (cm)
1	√				√		√		√	√		87.92	2.14	6.76
2		√			√		√		√	√		87.86	2.16	6.75
3			√		√		√		√	√		88.48	2.16	6.77
4			√	√			√		√	√		86.01	2.18	6.77
5			√		√	√			√	√		88.48	2.18	6.79
6			√		√			√	√	√		87.49	2.14	6.73
7			√		√		√			√		83.61	2.10	6.68
8			√				√		√		√	88.11	2.14	6.80

**Table 5:** Ablation study on 3DLoMatch with FPFH descriptor.

	NC	DD	SD	FM	SM	IC	MAE	MSE	RS	SVD	W-SVD	RR (%)	RE (°)	TE (cm)
1	√				√		√		√	√		44.47	3.89	10.25
2		√			√		√		√	√		43.91	3.93	10.33
3			√		√		√		√	√		44.64	3.91	10.39
4			√	√			√		√	√		37.79	3.85	9.89
5			√		√	√			√	√		43.68	3.96	10.33
6			√		√			√	√	√		44.41	3.93	10.24
7			√		√		√			√		40.37	3.90	10.20
8			√				√		√		√	44.24	3.90	10.11

**Table 6:** Comparison of model selection/recall in selection of different sizes of clique-like models under 3DMatch and 3DLoMatch datasets. $N_{n}$: Number of nodes. AS: Average selection. AR: Average recall.

$N_{n}$	3DMatch S/R (%)		3DLoMatch S/R (%)		AS (%)	AR (%)
	FPFH	FCGF	FPFH	FCGF		
20	30.87/27.30	7.89/6.22	30.77/13.92	21.50/11.85	22.83	64.54
15	24.77/22.06	7.52/6.90	27.23/12.97	20.27/9.38	18.86	67.60
10	19.53/17.13	11.71/10.29	21.79/9.77	19.82/11.51	18.65	64.88
5	14.73/13.00	23.04/21.50	13.03/5.73	22.46/14.43	18.54	72.82
3	10.10/9.00	49.85/48.98	7.19/2.25	15.95/12.75	21.12	84.01

**Table 7:** Model optimization on 3DMatch dataset with FPFH/FCGF. CO: Clique-like optimization. RO: Reselection optimization.

	CO	RO	RR (%)	RE (°)	TE (cm)	Time (s)
CL-PCR			88.48/93.90	2.16/2.04	6.77/6.54	0.51/0.73
Fast-CL-PCRv1	√		88.48/94.21	2.18/2.04	6.84/6.53	0.15/0.16
Fast-CL-PCRv2		√	88.54/93.78	2.16/2.03	6.77/6.49	0.24/0.10
Faster-CL-PCR	√	√	88.48/93.90	2.18/2.04	6.83/6.52	0.09/0.04

**Table 8:** Model optimization on 3DLoMatch dataset with FPFH/FCGF.

	CO	RO	RR (%)	RE (°)	TE (cm)	Time (s)
CL-PCR			44.64/59.91	3.91/3.67	10.39/10.43	0.35/0.42
Fast-CL-PCRv1	√		44.13/61.93	3.92/3.75	10.27/10.48	0.21/0.11
Fast-CL-PCRv2		√	44.64/60.64	3.91/3.67	10.40/10.30	0.35/0.23
Faster-CL-PCR	√	√	44.13/61.48	3.92/3.71	10.27/10.36	0.20/0.07

**Table 9:** Registration recall on 3DMatch and 3DLoMatch with FPFH and FCGF setting based on judging our/SC^2^-PCR++ [24]’s hypotheses. Success-n: a point cloud pair is considered alignable if at least n hypotheses are correct.

	3DMatch RR (%)		3DLoMatch RR (%)	
	FPFH	FCGF	FPFH	FCGF
success-1	96.00/91.50	98.34/96.80	70.19/49.35	87.37/77.04
success-5	90.02/80.10	97.23/93.84	69.12/24.99	74.68/65.47
success-10	86.01/72.34	96.18/92.11	76.16/15.27	68.78/57.89
success-20	79.11/59.03	94.82/89.28	72.80/6.68	61.48/48.29
success-50	68.02/36.29	91.56/81.52	50.52/0.95	47.89/33.46

**Table 10:** Comparison of our/SC^2^-PCR++ [24] with different overlap rates on real datasets.

Data	Error and Time	Overlap Rates					
		1	0.8	0.6	0.4	0.2	0.1
parts (a)	RE (°)	0.06/1.03	0.14/0.93	0.11/1.10	0.08/0.35	0.51/4.31	0.24/3.38
	TE (cm)	0.04/1.27	0.15/0.77	0.11/0.85	0.06/0.49	0.46/6.21	0.26/1.61
	PCR_time (s)	0.13/0.25	0.14/0.23	0.12/0.23	0.12/0.22	0.10/0.22	0.10/0.22
parts (b)	RE (°)	0.23/0.83	0.21/0.39	0.22/1.60	0.67/1.98	1.07/2.31	0.55/4.90
	TE (cm)	0.33/1.73	0.20/0.42	0.12/1.49	0.50/0.78	1.92/4.26	0.46/5.08
	PCR_time (s)	0.14/0.25	0.14/0.25	0.14/0.24	0.12/0.24	0.12/0.21	0.11/0.21
parts (c)	RE (°)	0.24/0.72	0.09/2.43	0.48/1.97	0.88/1.50	2.42/3.81	1.53/11.30
	TE (cm)	0.23/0.64	0.08/1.98	0.38/1.79	0.37/0.81	1.40/3.73	1.23/4.89
	PCR_time (s)	0.23/0.26	0.12/0.26	0.14/0.24	0.12/0.25	0.10/0.23	0.10/0.23

## Data Availability

Data underlying the result presented in this paper are available in Refs. [22,24]. Other generated data are not publicly available at this time but may be obtained from the authors upon reasonable request.
